# Identification of Novel Pyroptosis-Related Gene Signatures to Predict Prostate Cancer Recurrence

**DOI:** 10.3389/fonc.2022.814912

**Published:** 2022-05-20

**Authors:** Chun Li, Jie Zhu, Hexi Du, Chaozhao Liang

**Affiliations:** ^1^ Department of Urology, The First Affiliated Hospital of Anhui Medical University, Hefei, China; ^2^ Department of General Surgery, The First Affiliated Hospital of Anhui Medical University, Hefei, China; ^3^ Institute of Urology, Anhui Medical University, Hefei, China; ^4^ Anhui Province Key Laboratory of Genitourinary Diseases, Anhui Medical University, Hefei, China; ^5^ Central Hospital Affiliated to Shandong First Medical University, Jinan, China

**Keywords:** pyroptosis, prostate cancer, recurrence, immunity, prognosis

## Abstract

Prostate cancer (PCa) is a common malignant type of urogenital tract tumor with poor prognosis. Despite therapeutic advances, the recurrence and mortality rates of PCa have continued to increase with poor prognoses. Pyroptosis, also known as inflammatory cell necrosis, is a recently identified type of programmed cell death that can regulate the invasiveness, differentiation, proliferation, and metastasis of tumor cells; thus, it has a profound effect on the prognosis of patients with tumors. However, the relationship between pyroptosis and PCa remains unclear. We first identified 25 pyroptosis-related genes (PRGs) that were differentially expressed between PCa tissues and matched normal tissues in The Cancer Genome Atlas (TCGA) cohort. Based on the expression levels of 25 PRGs, PCa patients were clearly divided into two clusters and 17 PRGs were found to be significantly different between the two clusters, suggesting probable roles for these genes in the progression and recurrence of PCa. Therefore, the GSE40272 dataset with recurrence follow-up information was used to verify their value. Univariate analysis suggested that 5/17 genes were associated with recurrence, the number of genes did not decrease after least absolute shrinkage and selection operator (LASSO) regression analysis, and 5 PRGs constituted the risk score formula. Low-risk and high-risk subgroups identified using the recurrence model showed different disease-free survival (DFS) times (P<0.001) and the risk score of five PRGs was a factor of independence for recurrence in patients with PCa. In addition, Gene Ontology (GO) and Kyoto Encyclopedia of Genes and Genomes (KEGG) analyses suggested that these pathways, and comprising PRGs might be closely related to carcinogenesis and invasion of tumors, tumor microenvironment, and immune response. In conclusion, the expression signatures of PRGs play an important role in predicting PCa recurrence.

## Introduction

Prostate cancer (PCa) is the most malignant tumor of the male genitourinary tract, with approximately 1,276,106 new cases of PCa worldwide and a total of 358,989 deaths in 2018, ranking second in the incidence of male malignant tumors and posing a serious threat to the health of elderly men (1). With the spread of awareness regarding prostate-specific antigen (PSA) screening and health examination, more patients have access to radical local treatment but 30–40% of patients with PSA still show recurrence after local treatment or transfer. In medium–high risk patients, the biochemical recurrence rate within 5 years after radical prostatectomy is more than 50%, suggesting the need for attention to residual tumors after treatment, metastasis before treatment, or new therapeutic strategies to compensate for the lack of local treatment (2–4). Genetic biomarkers have shown potential for predicting PCa recurrence; however, these have not yet been used in medical practice and are only in the molecular research stage. Therefore, it would be of great significance to discover the prognostic or genetic characteristics associated with the recurrence of PCa.

In 2001, scientists proposed the concept of pyroptosis, describing it as a new type of programmed inflammatory cell death that triggers certain inflammatory bodies by activating inactive factors and lysing gasdermin D, leading to a variety of diseases, such as heart disease, stroke, microbial infections, and tumors (5–7). The relationship between pyroptosis and cancer is extremely complex. Although pyroptosis inhibits tumorigenesis and tumor progression, it also creates a microenvironment that delivers nutrients to the tumor and accelerates its growth (8). Studies have increasingly shown that pyroptosis affects the invasiveness, differentiation, proliferation, and metastasis of tumor cells, thereby affecting the prognosis of tumors (9–11). In addition, several studies have suggested that pyroptosis may be associated with regulation of the tumor immune microenvironment (12, 13).

In this study, pyroptosis likely had both positive and negative effects on PCa development. To date, the function of PRG expression in the prognosis of PCa is still unclear and none of the previous publications comprehensively evaluated PRGs in PCa. Moreover, the prognostic value of pyroptosis in PCa has not been reported. Therefore, studying the effect of pyroptosis on tumorigenesis and the development of PCa can facilitate: the evaluation of prognosis and recurrence in patients, a better understanding of the progression and metastasis of PCa, along with better guidance for the identification of new therapeutic targets. In the present study, we performed a comprehensive evaluation of differentially expressed PRGs in PCa and identified PRG-based differences to predict the recurrence of PCa, which may provide a new approach for its diagnosis and treatment.

## Materials and Methods

### Datasets

**Figure 1:**
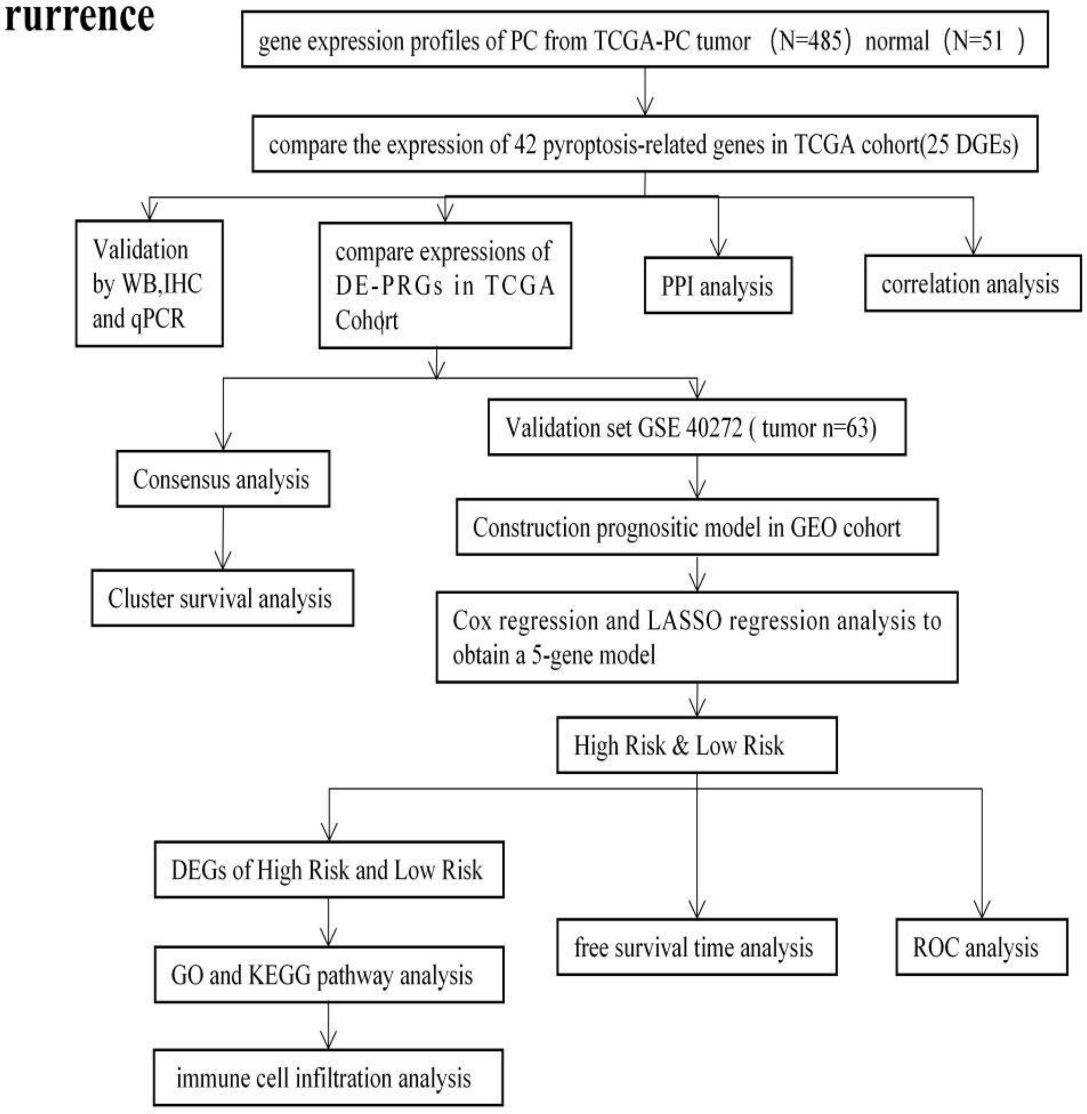
The flowchart of the overall procedures. This flowchart illustrates the process of data collection and analysis for prognostic study.

The design process and grouping are illustrated in **Figure 1**. The RNA sequencing (RNA-Seq) data of PCa patient samples, matched normal samples, and corresponding clinical features were obtained from the TCGA database (https://portal.gdc.- cancer.gov/, **Table 1**). Validation cohort of RNA-seq data and clinical information were from the GEO database on August 16, 2021 (https://ncbi.nlm.nih.gov/geo/, ID: GSE40272, **Table 1**).

**Table 1:** Clinical characteristic of patients with prostate cancer.

	TCGA	GEO
	**Number of patients**	
**Age**		
<60	283	25
≥60	202	38
**Stage**		
T TI-T2	187	–
T3-T4	296	
unknown	7	
N N0	340	–
Nl	78	
Unknown	72	
M	unknown	–
**Disease state**		
Dlive	476	–
Dead	9	
**Disease free survival**	–	63
**Treatment**		
Operation	–	62
Operation and hormone	–	1
	**Time**	
**Median disease free survival time**	–	35.82 months
**Median follow-up survival time**	1092.89 days	–

### Identification of DE-PRGs Between PCa and Matched Normal Control Groups

From the Reactome database (https://reactome.org/) and Molecular Signatures Database (https://www.gsea-msigdb.org/), 42 PRGs were obtained and verified in several reviews (13–20). TCGA expression data were uniformly standardized to fragments per kilobase per million (FPKM) prior to comparison. The 25 DE-PRGs were identified using the “limma” package, and the P value threshold was less than 0.05. The 25 DE-PRGs were annotated as follows: * P < 0.05, ** P < 0.01, and *** P <0.001. A protein–protein interaction (PPI) network was modelled using the Search Tool for the Retrieval of Interacting Genes/Proteins (STRING) version 11.0.

### Cell Lines and Cell Culture

PCa cell lines PC3, C4-2, 22RV1 and human prostatic epithelial cells (RWPE-1) were purchased from ATCC (American Type Culture Collection, Manassas, VA, USA). All PC cell lines were cultured in RP1640 medium (RP1640, Gibco) supplemented with 10% foetal bovine serum (FBS, Gibco), 100 U/mL penicillin, and 100 μg/mL streptomycin at 37°C in 5% CO2.

### Reagents and Antibodies

Reagents and antibodies used were: Cytochrome C antibody (AF0146) (Affinity Biosciences,USA); anti-GSDMB antibody (ab235540) and anti-caspase-8 antibody (Abcam, USA); and Bak (BAK1) antibody (AB016), BAX antibody, and TP53 antibody (AF1270) (Beyotime, China).

### Western Blotting

Cells were lysed in cold RIPA buffer (Beyotime, China) in the presence of 1 × protease inhibitor cocktail and 1 × PhosStop (Roche, Isere, France) after two washes with phosphate-buffered saline (PBS). The lysate was removed by sonication, and the protein concentration was determined using a Pierce bicinchoninic acid (BCA) protein assay kit (Thermo Fisher Scientific, MA, USA). Equal amounts of proteins were loaded onto polyacrylamide gels.

### Immunohistochemistry

Prostate tissue blocks were cut into 5-μm-thick sections, dewaxed in xylene, and rehydrated in an ethanol gradient. Antigen was retrieved by boiling the tissue sections for 20 minutes in retrieval buffer. Sections were later immersed in a 3% hydrogen peroxide solution for 15 minutes to block endogenous peroxidase activity. Next, the slides were rinsed with PBS 3 times, blocked with 3% BSA at room temperature for 30 minutes, and then incubated with purified rabbit anti-human primary antibody (1:300 dilution) at 4°C overnight. After incubation, the slides were incubated with diluted goat anti-rabbit secondary antibody for 1 hour at room temperature. They were then rinsed twice with PBS. The detection reagent DAB was added and the slides were incubated in the dark at room temperature for 10 minutes. After DAB staining, the slides were rinsed in running tap water for 3 minutes. Finally, they were incubated with haematoxylin to counterstain the nucleus. All slides were independently examined by two authorized pathologists who were not informed of the patients’ clinical statuses or outcomes.

#### RNA Extraction and Quantitative Analysis

##### Real-Time PCR (qRT–PCR)

Total RNA was extracted from cell lines with TRIzol Reagent (Invitrogen, USA). Total RNA was reverse-transcribed into cDNA with PrimeScript RT Master Mix (Takara, USA) and then used to perform quantitative real-time PCR (qRT–PCR) with SYBR qPCR Master Mix (Vazyme, China). GAPDH was used as an internal control for gene quantification. The 2−ΔCT was calculated for every sample and normalised to GAPDH. The primer sequences used are shown in **Table 2**.

**Table 2:** Primer Sequences Used in the qRT-PCR Assay.

Primer	Sequence (5'-3'))
BAK-For	GTTTTCCGCAGCTACGTTTTT
BAK-Rev	GCAGAGGTAAGGTGACCATCTC
BAX-For	CCCGAGAGGTCTTTTTCCGAG
BAX-Rev	CCAGCCCATGATGGTTCTGAT
CASP8-For	TTTCTGCCTACAGGGTCATGC
CASP8-Rev	GCTGCTTCTCTCTTTGCTGAA
CYCS-For	CTTTGGGCGGAAGACAGGTC
CYCS-Rev	TTATTGGCGGCTGTGTAAGAG
GSDMB-For	TGATTGCCGTTAGAAGCCTTG
GSDMB-Rev	TCCCGTTGAGTCTACATTATCCA
TP53-For	CAGCACATGACGGAGGTTGT
TPS3-Rev	TCATCCAAATACTCCACACGC
GAPDH-For	GGAGCGAGATCCCTCCAAAAT
GAPDH-REv	GGCTGTTGTCATACTTCTCATGG

#### Ethics Approval and Consent to Participate

Fifteen cases of cancer tissues and eleven cases of paracancerous tissues were extracted at the Pathology Department of the First Affiliated Hospital of Anhui Medical University and all patients were clinically diagnosed with prostate cancer from February 25, 2022 to March 26, 2022. The project was approved by the Ethics Committee of the hospital and written informed consent was obtained from each patient who enrolled in the study (Reference number: Quick-PJ 20220320).

### Consensus Clustering Analysis of PRGs

Consensus clustering, a technique for combining multiple clusters into a more stable single cluster, was used to distinguish different pyroptosis correlation patterns associated with pyroptosis regulation using the k-means method. The quantity and stability of the clusters were determined using the consensus clustering algorithm in the ConsensusClusterPlus package. The chi-square test and R package “survival” were used to analyse the correlation between clusters and overall survival (OS), showing the results by Kaplan–Meier curves. The differential expression analysis of PRGs among different clusters was performed again, and 17 DE-PRGs were displayed in the form of heatmaps.

### Establishment and Validation of the PRG Model of Recurrence and Prognosis

Seventeen PRGs were found to be related to PCa progression. Since very few patients died in the TCGA cohort, it was difficult to select PRGs closely related to OS. Therefore, the GSE40272 dataset with disease-free survival (DFS) traits was used to construct a prognostic model. First, univariate Cox regression analysis was used to assess the association between DFS status and 17 PRGs to evaluate their prognostic value. With 0.05 as the cut-off value, 5/17 genes involved in recurrence were identified for further analysis. Subsequently, the LASSO Cox regression model (R package “glmnet”) was used to construct a refined recurrence prediction model. Finally, the 5 PRGs and their coefficients were reserved to determine the penalty parameter λ using the minimum standard. The risk scoring formula was as follows: PCa recurrence risk score $(\text{PRRS})=\sum_{\left(n=1\right)}^{i}Coefi*Xi$ expression level (*Coefi* indicates the coefficients, and *Xi* represents the standardised levels of gene expression). Patients with PCa from the GSE40272 cohort were divided into low- and high-risk groups according to the median risk score, and the DFS time of the two groups was compared using Kaplan–Meier analysis. On the basis of the 5-gene signature, principal component analysis (PCA) was used to assess the separability of the two groups according to the “prcomp” function. A three-year ROC curve was analysed using the “survival”, “survminer”, and “time ROC” R packages.

### GO and KEGG (Gene Set Enrichment) Analysis of the DEGs

Patients with PCa from the GEO cohort were classified into two groups based on the median risk score. According to the specific standard (|log2FC| ≥ 1 and P value < 0.05), the DEGs between the low- and high-risk groups were extracted. Based on these DEGs, the “cluster profiler” package was applied to enrich GO and KEGG pathways and the “GO plot” package was used to visualize the results.

### Immune Infiltration Analysis

Single-sample gene set enrichment analysis (ssGSEA) was performed using the “gsva” package to calculate the immune cell infiltration score and to assess the activity of immune-related pathways.

### Statistical Analysis

Univariate analysis of variance was used to compare the gene expression levels between matched normal prostate tissues and PCa tissues, while the Pearson chi‐square test was applied to compare the categorical variables. The Kaplan–Meier method was used to perform a bilateral log-rank test to assess the DFS of patients in the two subgroups. In addition, a univariate Cox regression model was used to evaluate the recurrence value of this risk model. The infiltration of immune cells and activation of immune pathways were compared between the two groups and the Mann–Whitney test was performed. R software (v4.0.2) was used to perform all statistical analyses.

## Results

### Identified DEGs From Matched Normal and Cancer Tissues in the TCGA Cohort

**Figure 2:**
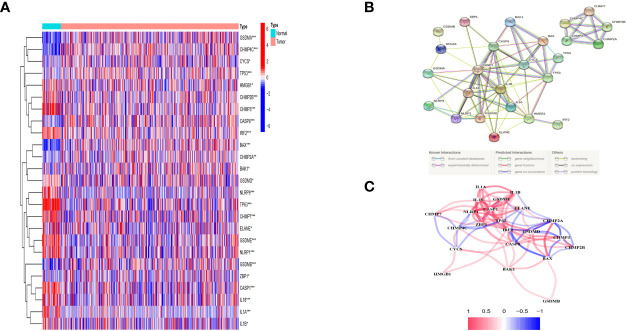
Expressions of the 25 differentially expressed pyroptosis-related genes between tumor and normal and the interactions among them. **(A)** Heatmap (green: low expression leveI; red; high expression level) of the pyroptosis-related genes between the normal (N, brilliant blue) and the tumour tissues (T.red). P values were showed as: *P < 0.05; **P < 0.01; ***P < 0 001. **(B)** PPl network showing the interactions of DEPRGs (interaction score= 0.9). **(C)** The correlation network of DEPRGs (red line: positive correlation: blue line negative correlation. The depth of the colours reflects the strength of the relevance).

The expression levels of 42 PRGs were detected in 485 tumor tissues and matched normal tissues from the TCGA database and 25 DE-PRGs were identified (P < 0.05). Among them, 10 genes (*BAK*, *BAX*, *CASP8*, *CHMP2A*, *CHMP4C*, *CSCY*, *GSDMA*, *GSDMB*, *TP53*, and *ZBP1*) were upregulated, whereas 15 genes (*CASP1*, *CHMP2B*, *CHMP3*, *CHMP7*, *ELANE*, *GSDMD*, *GSDME*, *HMGB1*, *IL18, IL* ***α*** *, ILβ*, *IRF2*, *NLRP1*, *NLRP9*, and *TP63*) were downregulated in the cancer tissues. The RNA expression profile of the DEGs is shown in **Figure 2A** (red and blue colors indicate higher and lower expression levels, respectively). **Figures 2B, C** shows the PPIs and related network of DE-PRGs in TCGA data, where the interaction score was 0.9 (the highest confidence). The results showed that *BAK1*, *BAX*, *CASP8*, *CASP1*, *IL18*, *IL1β*, *CYCS*, *GSDMB, GSDMD and TP53* are hub genes.

### Validation of the Hub DE-PRGs by Western Blotting and Immunohistochemical Staining

**Figure 3:**
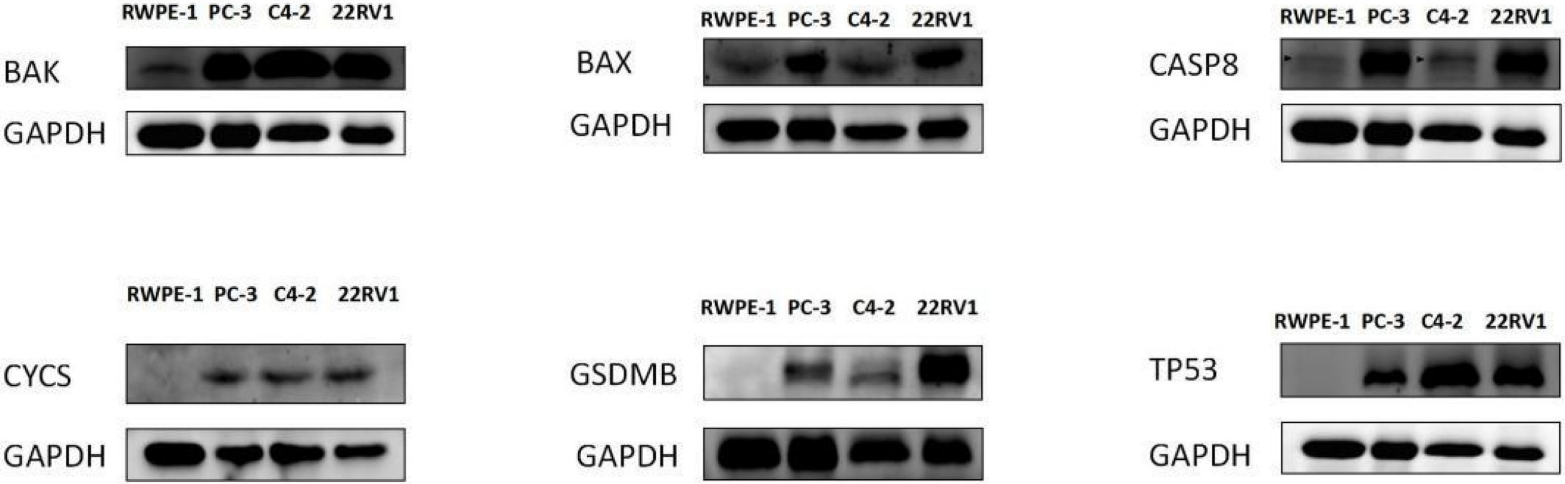
WESTERN-BLOT validation of hub DE-PRGs expressions in normal and tumor tissues, the results showed that protein expression levels of CASP8, BAK, BAX, CYCS, TP53, and GSDMB genes were highly expressed in three CRPC (Castration Resistant Prostate Cancer) cell lines compared to the control normal cell line (RWPE- I ). CRPC cell lines (PC-3, C4-2, 22RVI), Control cell line (normal human prostatic epithelial cell, RWPE-1).

**Figure 4:**
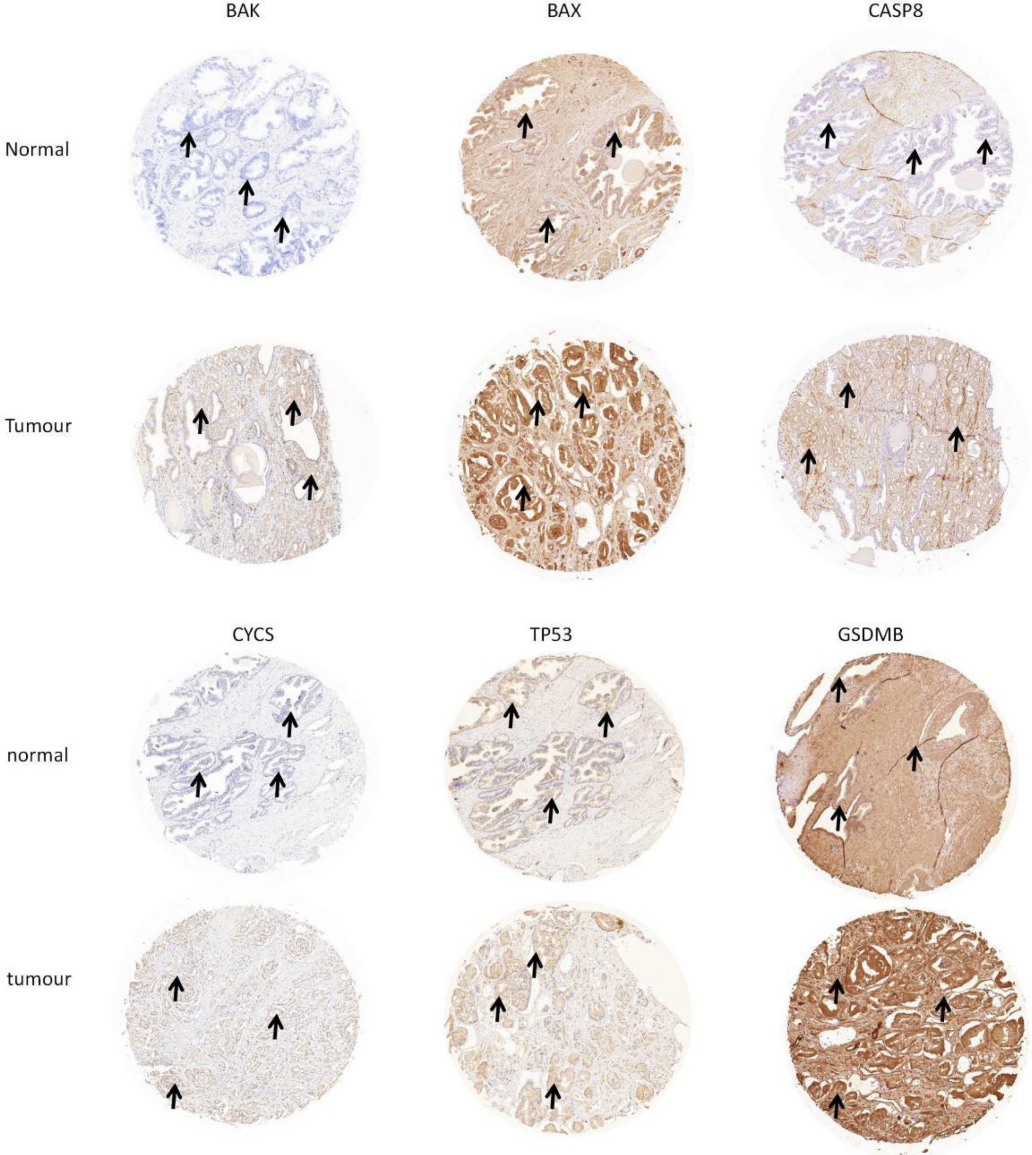
Verification of hub DE-PROs expressions in normal and tumour tissue with lmmunohistochemistry (lHC), the expression of glandular epithelium (cytoplasm, cell membrane) in tumour group was significantly higher than that in normal group (obviously deepened in yellowish brown compared to normal groups. “ →”).

Western blotting was used to validate the expression levels of differentially expressed pyroptosis-related genes, including *CASP8, BAK, BAX, CYCS, TP53, and GSDMB* in three castration-resistant prostate cancer (CRPC) cell lines (PC3, C4-2, and 22RV1) and one normal human prostatic epithelial cell line (RWPE-1). The results showed that the protein expression levels of *CASP8, BAK, BAX, CYCS, TP53, and GSDMB* were higher in the three CRPC cell lines than in the control cell line (RWPE-1). Subsequently, immunohistochemical validation of human tissues was performed. This finding was consistent with our prediction (**Figures 3**, **4**). The results showed that the expression of glandular epithelium (cytoplasm, cell membrane) in the tumor group was significantly higher than that in the normal group (clearly deepened yellowish brown compared to normal groups). All pathological tissues and immunohistochemical sections were confirmed by two senior pathologists.

### Validation of the Hub DE-PRGs Genes by qRT-PCR

**Figure 5:**
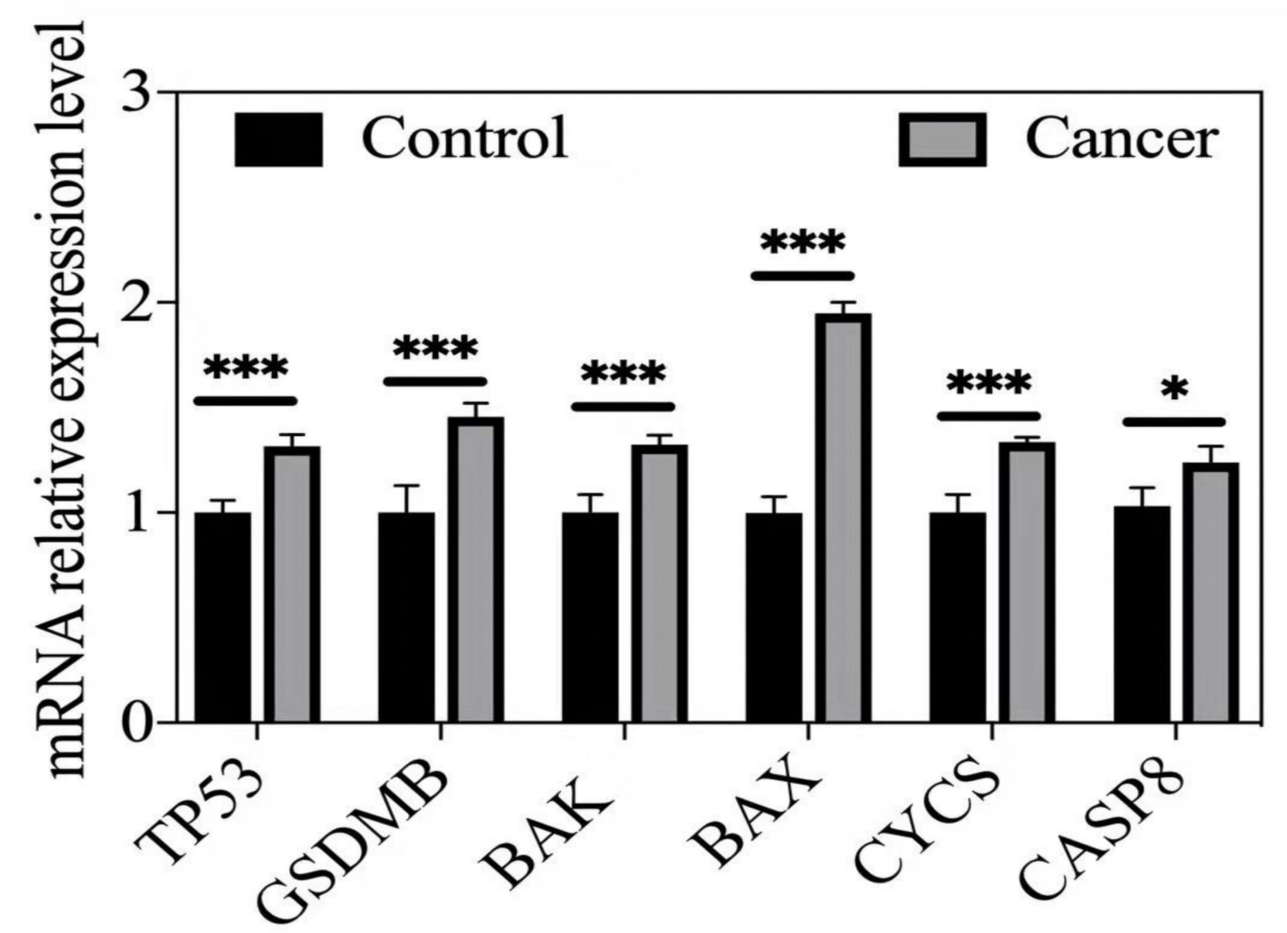
qRT-PCR validation or hub DE-PRGs in human prostate cancer tissues compared to control tissues. *p < 0.05; ***p < 0.001. Cancer (n = 5-6), human prostate cancer tissues. Control (n = 5-6), matched human normal prostate tissues.

qRT–PCR was used to validate the expression of the hub DE-PRG genes, including *CASP8, BAK, BAX, CYCS, TP53, and GSDMB* in human prostate cancer tissues and matched normal prostate tissues. The results show that the *CASP8, BAK, BAX, CYCS, TP53, and GSDMB* genes were highly expressed in human prostate cancer tissues compared to matched normal prostate tissues. This is consistent with our predictions. The results are shown in **Figure 5**.

### Tumor Classification Based on the DE-PRGs in the TCGA Cohort

**Figure 6:**
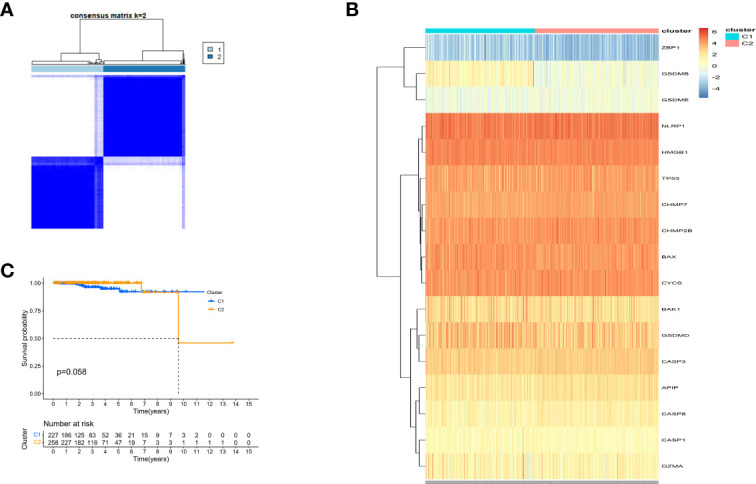
Tumour classification based on the pyroptosis-related DEGs. **(A)** 485 PC patients were grouped into two clusters according to the consensus clustering matrix (k=2). **(B)** Heatmap and the clinicopathologic characters of the two clusters classified by these DEPRGs. **(C)** Kaplan-Meier OS curves for the two clusters.

Consensus clustering analysis was performed on all 485 patients with PCa in the TCGA cohort to study the relationship between the expression of 25 DE-PRGs and PCa subtypes. An unsupervised clustering method was used to identify two different regulatory patterns by increasing the clustering variate k from 2 to 10. When k was equal to 2, the intragroup and intergroup correlations were the highest and lowest, respectively, which could be well classified into two clusters (**Figure 6A**). The heatmap shows the DE-PRGs between the two clusters (**Figure 6B**). Subsequently, the OS time between the two clusters was compared and no significant differences were found (P = 0.058, **Figure 6C**).

### Construction of the Prognostic Model Based on DE-PRGs in a GEO Cohort

**Figure 7:**
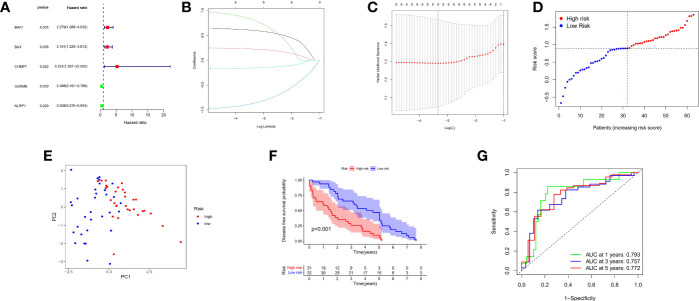
Construction of risk signature in the GEO cohort. **(A)** Univariate cox regression analysis of DFS for each pyroptosis-related gene, and genes with P < 0.05. **(B)** LASSO regression of the 5 DFS-related genes. **(C)** Cross-validation for tuning the parameter selection in the LASSO regression. **(D)** Distribution of patients based on the risk score. **(E)** PCA plot for PCs based on the risk score. **(F)** Kaplan-Meier curves for the DFS of patients in the high- and low-risk groups. **(G)** ROC curves demonstrated the predictive sensitivity of the risk score.

Information on 63 patients with PCa was obtained from the GEO database (GSE40272), and the data were randomized. Five out of seventeen genes (*BAK*, *BAX*, *CHMP7*, *GSDMB*, and *NLRP1*) met the standard of P value < 0.05 by univariate Cox regression analysis. Among these, three genes (*BAK1*, *BAX*, and *CHMP7*) were related to increased risk in HR>1, while the other two genes (*GSDMB* and *NLRP1*) were associated with lower risk in HR<1 (**Figure 7A**). LASSO Cox regression analysis did not reduce the genes; thus, a 5-gene signature was constructed based on the optimum λ value (**Figures 7B, C**)and subsequently, a pyroptosis-related signature risk score known as the “PRRS” was built. The PRRS was calculated as PRRS = (0.598*BAK exp.) + (0.223*BAX exp.) + (0.800*CHMP7 exp.) + (-0.863*GSDMB exp.) + (-0.155*NLRP1 exp.). The high-risk group of 31 patients with PCa and the low-risk group of 32 patients with PCa were divided according to the GEO cohort median risk score (**Figure 7D**). The PCA indicated the separation of satisfaction between the two groups (**Figure 7E**). Furthermore, a clear distinction in DFS was observed in the Kaplan–Meier analysis between these two groups (P value <0.001, **Figure 7F**). ROC analysis in the GEO cohort had a significant predictive effect on PCa recurrence (1-year AUC = 0.793, 2-year AUC = 0.757, and 3-year AUC = 0.772) (**Figure 7G**).

### Functional Enrichment Analysis of DEGs

**Figure 8:**
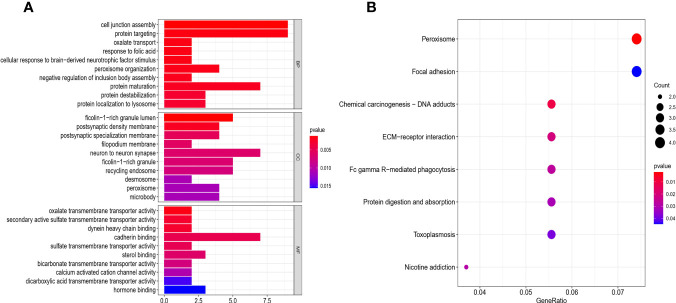
Functional analysis based on the DEGs between the two-risk groups in the GEO cohort. **(A)** Barplot graph for GO enrichment (the longer bar means the more genes enriched, and the increasing depth of red mans the differences were more obvious). **(B)** Bubble graph for KEGG pathways (the bigger bubble means the more genes enriched, and the increasing depth of red means the differences were more obvious; q-value: the adjusted p-value).

To identify other pathways that may be closely related to pyroptosis-related pathways, all DEGs (**Table S1**) of the two risk groups were analyzed for GO and KEGG enrichment. These significant DEGs were found in the major positive regulation of cell junction assembly, protein targeting, protein maturation, peroxisome organization, ficolin-1-rich granule lumen, neuron-to-neuron synapse, recycling endosome, and cadherin binding in GO analysis. Moreover, KEGG analysis showed that these DEGs were mainly involved in the regulation of Fc gamma R-mediated phagocytosis, peroxisomes, chemical carcinogenesis, ECM-receptor interaction, and focal adhesion (**Figures 8A, B**).

### Association between the Immune Status of Patients and PCa Risk in the GEO Cohort

**Figure 9:**
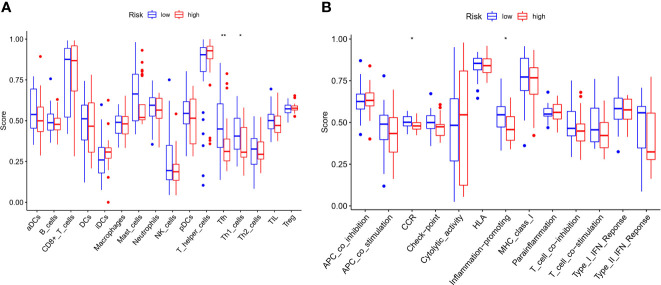
Comparison of the ssGSEA scores for immune cells and immune pathways. **(A)** Comparison of the enrichment score s of 16 types of immune cells between low- (blue box) and high-risk (red box) group in the GEO cohort. **(B)** Comparison of the and 13 immune-related pathways between low- (blue box) and high-risk (red box) group in the GEO cohort. P values were showed as: ns not significant; *P < 0.05; **P < 0.01.

We further explored the changes in immune cell infiltration between the low- and high-risk groups. Based on functional analysis, the activity of 13 immune-related pathways and the enrichment fractions of 16 types of immune cells were compared in the two risk groups and the results were compared using ssGSEA in the GEO cohort. The results showed that, the level of immune cell infiltration was generally lower in the high-risk subgroup, especially in Tfh and Th1 cells. Conversely, CCR and the inflammatory-promoting activity of immune pathways were lower in the high-risk group (**Figures 9A, B**).

## Discussion

Pyroptosis, mediated by the gasdermin family and associated with inflammatory and immune responses, is a recently discovered form of programmed cell death. The early stage of pyroptosis has always been considered an apoptotic process and the downstream pathway of pyroptosis activation by infection or injury has been elucidated. Recent evidence suggests that pyroptosis is related to cancer and the dual effects of pyroptosis have aroused the interest of researchers. However, the relationship between pyroptosis and PCa has not been elucidated. Pyroptosis may have two sides in cancer patients, acting as a double-edged sword. The most direct way to better understand pyroptosis and its importance is to establish a prognostic model.

In this study, we first clarified the expression and prognostic value of PRGs in PCa, studied the mRNA expression levels of 42 PRGs in PCa and matched normal tissues, and found 25 differentially expressed genes. To explore the relationship between the expression of these PRGs and PCa subtypes, we identified 17 pyroptosis-related DEGs associated with survival. Our findings suggested that these genes play an important role both in the pathogenesis of cancer and in the heterogeneity of cancer patients and we inferred that differential expression of these genes was related to a reduced death rate in the TCGA database. Therefore, to further evaluate the relationship of these PRGs with PCa survival, we searched the GEO database and other related databases and found insufficient follow-up survival data on PCa; however, a set of follow-up data of recurrence (DFS) rate caught our attention. Although not significant in the survival analysis, the high recurrence rate of primary PCa may be valuable. Therefore, we aimed to verify the relationship between these PRGs and recurrence. In addition, we evaluated the prognostic value of these pyroptosis-related regulatory factors and obtained the risk profile of five genes using univariate Cox and LASSO Cox regression analyses with the GEO cohort. The results showed that these pyroptosis-related risk genes constitute a risk formula to predict the recurrence of patients, which is similar to a biomarker: the higher the risk score is, the worse the recurrence.

In our study, ten hub genes were identified in the PPI network. However, how these PRGs interact and whether they are relevant to patient prognostic outcomes remain unclear. For example, Casp1 encodes *CASP1*, a member of the caspase family that is activated by inflammasomes and induces pyroptosis (21). Casp1 is underexpressed in a variety of tumor tissues when it acts as a tumor suppressor (22). Based on our differential gene sorting analysis, it was observed to be poorly expressed in tumors, which is consistent with previous reports. Casp8 can cause pyroptosis by cleaving GSDMD into its active form when TAK1 is suppressed, and TAK1 inhibition also leads to GSDME cleavage (23). In addition, activation of Casp8 can drive inflammasome-independent IL-1β and exogenous cell death receptor signaling downstream of GSDMD to convert apoptotic signals into GSDMD-dependent pyroptosis-like cell death (24). Both our prediction and experimental validation showed that Casp8 was elevated, suggesting that we can treat PCa by activating the Casp8 pathway to trigger cell death in the future. There is an opportunity to further verify this. BAK and BAX are well-known regulators of the apoptosis pathway and have been found to play a role in the pyroptotic pathway. As tumors grow and progress, these pathways inevitably interact. Studies have shown that the Bak/Bax-Caspase-3-GSDME pathway can enhance the antitumor effect (25). Similar to CASP8, BAK and BAX were elevated in our prediction and validation and were important components of the prediction formula. We hypothesized that BAK and BAX could be used to treat high-risk recurrent PCa by coactivating upstream and downstream genes to promote pyroptosis and kill tumors. Furthermore, interleukin IL1β, a proinflammatory factor that induces pyroptosis (26), has a tumorigenic effect, and its main role is to promote proliferation, migration, and metastasis (27). GSDMB belongs to the GSDM family and is more widely expressed than other members of the GSDM family. Pyroptosis is induced by the cleavage of GSDMB by lymphocytic granzyme A (28). This study demonstrates that activation of GSDMB induces pyroptosis and promotes tumor clearance, supporting an important regulation of reactivation in our predicted formula with a high-risk factor. NLRP1 is an NLR family protein that can also induce apoptosis and pyroptosis, which can impact cancer pathogenesis by modulating congenital immune responses, dysregulation of NLR family members, and results in various inflammatory diseases and autoimmune disorders. Studies have shown that the mRNA and protein levels of NLRP1 are reduced in colorectal cancer cells compared to normal cells and the anticancer drug DAC increases the expression of NLRP1 to inhibit the progression of colorectal cancer (29, 30). In our differential pyroptosis gene expression and prediction formula, NLRP1 was identified as a low-risk factor, which was consistent with literature reports. Immunohistochemistry showed that NLRP1 expression was lower in the high-grade PCa group than in the low-grade PCa group. According to our results, some of these PRGs appear to be cancer suppressor genes because they are downregulated threefold in cancer tissues, Nevertheless, they also help prolong patients’ DFS because they are enriched in the low-risk group. Additionally, five genes were identified as promoters of pyroptosis in the prognostic model. However, not all of these promoters were related to better PCa prognosis in our study, indicating that these five genes are in the same formula and their sensitivity and specificity are mutually restricted to achieve the optimal ROC. Therefore, it was not possible to determine the expression level of one gene separately to evaluate high or low risk. We will further study how these genes interact with each other during pyroptosis.

To identify other pathways that might be closely related to pyroptosis-related pathways, all DEGs (**Table S1**) in the two risk groups were analyzed for GO and KEGG enrichment. The results showed that these genes were mainly involved in regulatory protein targeting, cadherin binding, chemical carcinogenesis, ECM-receptor interaction, and focal adhesion. Studies have shown that cadherin-11 affects the invasiveness and migration of PCa cells (31–34). These functions or pathways suggest that PRGs might play an important role in the oncogenesis, recurrence, and metastasis of PCa and may be related to the intensity of pyroptosis or counter-regulation.

Another important finding of our study was that PRGs were correlated with immune infiltration, and 13 of 16 important antitumor immune cells were increased in the low-risk group compared to the high-risk group. However, some comparisons were not significantly different, probably because of the limited number of samples in the two cohorts. In the GEO cohort, the accumulation of cancer-promoting immune cells in the tumor microenvironment was generally observed in the high-risk group. Tfh and Th1 cells were statistically significant in our study. Relevant studies have shown that blood Th1 levels are negatively correlated with PCa and can reduce the occurrence of prostate bone metastasis and improve survival. Notably, Th1 levels in the high-risk group were lower than those in the low-risk group and exhibited a potential immunotherapeutic effect, which is consistent with our findings (35–37). Based on these results, poor DFS in high-risk PCa patients might be related to the suppressed levels of antitumor immunity and changes in the tumor microenvironment.

The advantage of our study lies in the systemic analysis based on TCGA and GEO cohorts and the evaluation of PRGs in PCa. Our study has several limitations. Owing to the nature of publicly available datasets, the number of deaths among patients with PCa was very limited. The specific mechanism by which PRGs regulate PCa occurrence and progression remains to be explored. Large and well-designed clinical or *in vivo* experiments are required to validate our predictive model. Despite these limitations, our experiments achieved some consistency with the predictions. In summary, we conducted a comprehensive and systematic bioinformatics analysis and identified PRG signatures that were significantly associated with DFS in patients with PCa and some relevant experiments were performed to verify our results. Furthermore, the risk score based on the prognostic model of five PRGs was an independent risk factor for PCa recurrence and was found to be related to the immune microenvironment, which should be verified in future studies.

## Data Availability Statement

The datasets presented in this study can be found in online repositories. The names of the repository/repositories and accession number(s) can be found in the article/**Supplementary Material**.

## Ethics Statement

The studies involving human participants were reviewed and approved by Clinical Medical Research Ethics Committee of the First Affiliated Hospital of Anhui Medical University. The patients/participants provided their written informed consent to participate in this study.

## Author Contributions

CL and JZ conceived the study. CL and JZ designed the study and analyzed the data. CL wrote the manuscript, which was reviewed by CZL and HXD. All authors contributed to the article and approved the submitted version.

## Funding

This work is supported by the National Natural Science Foundation of China (31800834, 81773299, 82072055, 82072040, 82100815), and Anhui Natural Science Foundation of China (2108085QH315).

## Conflict of Interest

The authors declare that the research was conducted in the absence of any commercial or financial relationships that could be construed as a potential conflict of interest.

## Publisher’s Note

All claims expressed in this article are solely those of the authors and do not necessarily represent those of their affiliated organizations, or those of the publisher, the editors and the reviewers. Any product that may be evaluated in this article, or claim that may be made by its manufacturer, is not guaranteed or endorsed by the publisher.
